# Deep-Ultraviolet (DUV)-Induced Doping in Single Channel Graphene for Pn-Junction

**DOI:** 10.3390/nano11113003

**Published:** 2021-11-09

**Authors:** Asif Ali, So-Young Kim, Muhammad Hussain, Syed Hassan Abbas Jaffery, Ghulam Dastgeer, Sajjad Hussain, Bach Thi Phuong Anh, Jonghwa Eom, Byoung Hun Lee, Jongwan Jung

**Affiliations:** 1 HMC (Hybrid Materials Center), Department of Nanotechnology and Advanced Materials Engineering, Sejong University, Seoul 05006, Korea; asifmju@gmail.com (A.A.); mhussain@sju.ac.kr (M.H.); hassam@sju.ac.kr (S.H.A.J.); shussainawan@gmail.com (S.H.); bachthiphuonganh@gmail.com (B.T.P.A.); 2 Center for Semiconductor Technology Convergence, Department of Electrical Engineering, Pohang University of Science and Technology, Cheongam-ro 77, Nam-gu, Pohang 37673, Korea; una0918@postech.ac.kr (S.-Y.K.); bhlee1@postech.ac.kr (B.H.L.); 3 Department of Physics & Astronomy, Graphene Research Institute-Texas Photonics Center International Research Center (GRI–TPC IRC), Sejong University, Seoul 05006, Korea; gdastgeer@sejong.ac.kr (G.D.); eom@sejong.ac.kr (J.E.)

**Keywords:** graphene, DUV irradiation, p-doping, n-doping, pn-junction

## Abstract

The electronic properties of single-layer, CVD-grown graphene were modulated by deep ultraviolet (DUV) light irradiation in different radiation environments. The graphene field-effect transistors (GFETs), exposed to DUV in air and pure O_2_, exhibited p-type doping behavior, whereas those exposed in vacuum and pure N_2_ gas showed n-type doping. The degree of doping increased with DUV exposure time. However, n-type doping by DUV in vacuum reached saturation after 60 min of DUV irradiation. The p-type doping by DUV in air was observed to be quite stable over a long period in a laboratory environment and at higher temperatures, with little change in charge carrier mobility. The p-doping in pure O_2_ showed ~15% de-doping over 4 months. The n-type doping in pure N_2_ exhibited a high doping effect but was highly unstable over time in a laboratory environment, with very marked de-doping towards a pristine condition. A lateral pn-junction of graphene was successfully implemented by controlling the radiation environment of the DUV. First, graphene was doped to n-type by DUV in vacuum. Then the n-type graphene was converted to p-type by exposure again to DUV in air. The n-type region of the pn-junction was protected from DUV by a thick double-coated PMMA layer. The photocurrent response as a function of Vg was investigated to study possible applications in optoelectronics.

## 1. Introduction

The unique properties, such as ultrafast carrier dynamics, tunable optical properties by electrostatic doping, and gapless band structure, exhibited by graphene, have made it a promising candidate for multiple applications in electronic devices and optoelectronic devices [1,2,3,4]. The single-layer sp^2^–hybridized carbon atom graphene with one atom thickness has been intensively studied due its physical properties, including flexibility, thermal conductivity, mechanical strength, ballistic charge transport, high carrier mobility, and ambipolar transport. There are several methods to attain single-layer graphene, such as mechanical exfoliation of graphite, epitaxial growth of graphene, and chemical vapor deposition (CVD) on a metal (Cu or Ni) foil substrate [5,6,7,8]. The most suitable and promising process to obtain single-layer graphene on a larger scale is CVD growth of graphene on a metal (Cu or Ni) substrate [5,6,9].

One of the most important factors for the electronic and optoelectronic application of graphene is the engineering of its electrical properties and tuning of the Fermi level, by applying different doping methods [10,11,12]. Graphene, having a zero-band gap, requires a controlled doping method to modulate its electronic properties [10,11]. The Dirac equation describes the electron transport in graphene to explain the mobility of charge carriers [13,14]. Several graphene doping methods have been investigated in recent studies that include chemical doping, electrochemical doping, ion or electron beam irradiation, metal decoration or deposition, electrostatic doping, electrical stress-induced doping, absorption and desorption of gas molecules, and ultra-violet (UV) light illumination [14,15,16,17,18,19,20,21,22,23,24,25]. Graphene surface doping, without effect on the honeycomb structure which can be caused by other methods including chemical doping or the absorption and desorption of gas molecules, is usually unstable under experimental conditions and in a laboratory atmosphere [18,19,26]. There are many methods, including e-beam irradiation or plasma treatment of graphene, that can be applied to tune its electrical properties but these result in local defect formations in the graphene and affect its chemical properties [17,20,26]. Although there are some studies that have been reported on the carrier doping of the graphene by deep ultra-violet (DUV) irradiation, the effect of DUV on graphene is yet to be thoroughly explored [20,22,23,24,25,27]. The experimental conditions under which DUV irradiation is carried out can introduce either p or n-type stable or reversible doping to the graphene [20,22,23,24,25,27]. The coating of graphene with different materials and polymers that can absorb light is a useful way to reduce the effect on its properties [28].

The photo-assisted absorption or desorption of oxygen molecules, over pristine or functionalized CVD graphene, can introduce modulation in the doping level by tuning the Fermi levels. Under DUV light irradiation, the graphene reacts with the oxygen atoms that attach to the sites of pristine graphene and induce p-type doping [29]. It has been reported that UV-irradiation over graphene in air, or in an oxygen environment, can induce stable and reversible p-type doping [20,23,30]. The induction of n-type doping in graphene, treated under UV-irradiation in different gas environments (NH_3_, N_2_), by chemical coating (KnO_3_), or in vacuum, has also been reported [21,22,23,24,25,27,31]. The n-type doping effect of DUV irradiation in the presence of different gas environments is because the gas atoms are incorporated into the graphene lattice [21,22]. The UV-irradiation in vacuum generates dangling bonds at the graphene and SiO_2_ substrate interface which act as carrier traps in the channel; annealing removes them, resulting in an n-type doping effect in the graphene [24]. The exact mechanism accounting for n-type doping by DUV in vacuum is still unclear and requires further research, but the introduction of n-type doping has been attributed to electron trapping adsorbate groups on the graphene surface or graphene-substrate interface [24,25,32]. To investigate and understand the doping stability and doping effect of DUV irradiation on graphene, further experiments under controlled conditions and in defined environments are required. Stable n and p-doping in graphene have been utilized to fabricate graphene diodes and FETs for applications in electronic and optoelectronic devices [2,33,34,35].

In this study, we fabricated CVD-graphene field-effect transistors (GFETs) over an SiO_2_ substrate to investigate the effect of DUV irradiation on graphene doping under controlled experimental conditions. The fabricated devices were treated under DUV light irradiation in different environments such as air, pure O_2_ (99.995% pure), vacuum, and pure N_2_ (99.999% pure). Electrical transport measurements and Raman spectroscopy indicated the p-type doping behavior for the devices (GFETs) exposed to DUV in air and pure O_2_, and n-type doping for devices exposed in vacuum and pure N_2_. The stability of p-type and n-type doping, induced by DUV irradiation under different conditions, was studied throughout the heating time of the samples at 200 °C. Finally, a lateral pn-junction of graphene was successfully implemented by controlling the radiation environment of DUV. The gate-controlled photocurrent and photo response for graphene FET were measured. The GFET was observed to have photovoltaic effects, as has been observed in pn-junctions fabricated by other 2D materials [36]. The results of this study indicate that modulation of the electrical and photo-electrical properties of graphene FET and the formation of pn-junctions by DUV irradiation, under controlled experimental conditions, is a suitable and easily applicable approach for a range of technological, electronic, and photo-electronic applications.

## 2. Materials and Methods

**Figure 1:**
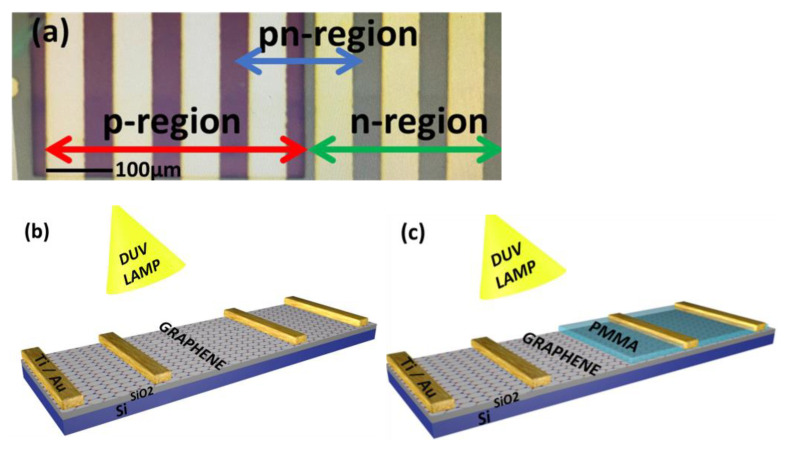
Optical microscope image and schematic figure: (**a**) The optical microscopic image of the fabricated device defining the p, n, and pn-junction regions of the GFET. (**b**) A schematic figure of the fabricated GFET device exposed to DUV irradiation under different environmental conditions. (**c**) A schematic figure of the pn-junction, fabricated on a single layer graphene channel of a GFET device by utilizing the DUV doping effect in air and vacuum and coating graphene partially by double PMMA layers.

A wet transfer method using PMMA was utilized to transfer graphene onto Cu foil, to a highly p-doped Si substrate with a 300 nm SiO_2_ surface layer. Figure 1 shows a schematic illustration and an optical microscope image of a GFET with a fabricated pn-junction. The active region, or Hall bar, of graphene on the substrate was made using photolithography and O_2_ plasma etching. The metal pads around the active region of graphene were formed by photolithography, and metal (Ti/Au of 5/40 nm) was deposited using thermal evaporation. The contact of metal pads to the graphene active region was made by e-beam lithography and Ti/Au (10/50 nm). All the device channel sizes were kept constant for the experiment, with a channel length of 5 nm. DUV light (λ = 220 nm, intensity = 11 mWcm^−^^2^) from a Bachur LS-100X-5 DUV exposure system was irradiated onto the samples from 20 cm in different environments (e.g., air, pure O_2_, pure N_2,_ and vacuum (~10^−4^ Torr)), with the graphene covered with PMMA layers, to study the doping effect induced in the graphene by DUV. The PMMA-A2 (1274 nm) layer was coated using a spin coating method with a speed of 4000 rpm. The Raman spectra of devices before and after different DUV treatment times for different environmental conditions were measured by a Renishaw micro-spectrometer (Seoul, South Korea) with a laser wavelength of 514 nm, over a range of 1100 to 3200 cm^−1^ wavenumbers. To reduce the local heating effect, the spot size of 1 um and power of 1.0 mW were kept fixed. The bulk carrier concentration, sheet carrier concentration, mobility, sheet resistance, and average Hall coefficient for p and n-type doped CVD-graphene were measured and confirmed by a four-probe Hall measurement system (HMS-3000, Ecopia, Anyang, South Korea). The electrical and photo-electrical measurements of the devices, under different DUV treatment conditions, were taken using a Keithley 4200A-SCS (Seoul, South Korea) semiconductor device analyzer.

## 3. Results and Discussions

**Figure 2:**
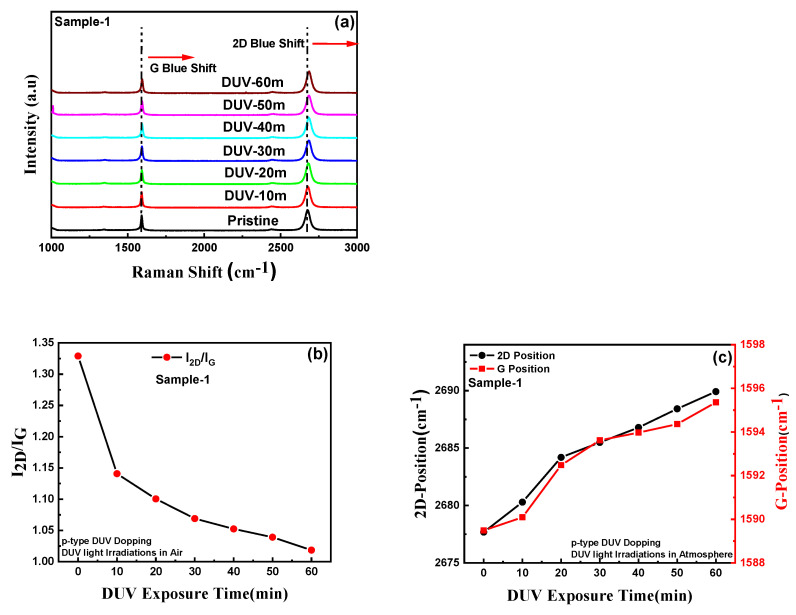
Raman spectroscopy analysis: (**a**) Raman spectra of pristine graphene and p-type doped sample-1 by DUV irradiation in air with different exposure times, showing the blue shift in 2D and G peaks towards higher wavenumbers. (**b**) A shift in the 2D and G peak intensity ratios with an increment in DUV exposure time. (**c**) The 2D and G peak positions as a function of DUV exposure time, showing the shift towards higher wavenumbers with an increase in the p-type doping effect by DUV irradiation in air.

**Figure 3:**
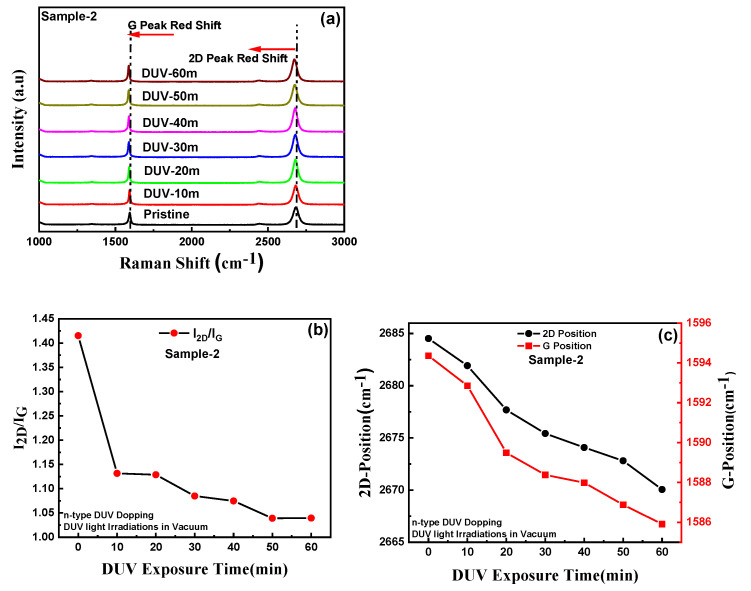
Raman spectroscopy analysis: (**a**) Raman spectra of pristine graphene and n-type doped sample-2, by DUV irradiation in vacuum with different exposure times, showing the redshift in 2D and G peaks towards smaller wavenumbers. (**b**) A shift in 2D and G peak intensity ratios with an increment in DUV exposure times. (**c**) The 2D and G peak positions as a function of DUV exposure times, showing the shift towards smaller wavenumbers with an increase in n-type doping effect by DUV irradiation in vacuum.

The optical microscope image of the graphene pn-diode is shown in Figure 1a and the schematic figure of the GFET and graphene pn-junction is presented in Figure 1b,c. Figure 2a shows the Raman spectra of single-layer graphene before and after DUV irradiation in air, for different exposure times (Sample-1). The blue shifts in the G and 2D peak positions can be observed for different DUV exposure times. Figure S1a shows the overlaid Raman spectrum for sample-1 with different exposure times to induce p-type doping in graphene. The E_2g_ optical phonon scattering at the Brillouin zone center is represented by the G peak and the scattering of two phonons with an opposite wave vector with momentum conservation is represented by the 2D peak, which is the second order of the D peak. This is termed the defect peak of graphene. The small D peak at around 1348.28 cm^−1^ is attributed to A_1g_ phonon symmetry near the boundary of the K-zone. The intensity of the D peak normally represents the number of defects in the graphene; the very low intensity in the graphene samples indicates that they were of good quality. It was also observed that the longer DUV exposure time did not readily change the intensity of the D peak. As reported previously, the characteristics of the G peak appear near 1589 cm^−1^ and the 2D peak at around 2677 cm^−1^; for our sample, the peaks of the pristine single layer CVD-grown graphene were observed at similar locations [23,37,38]. The Raman mapping, showing the G and 2D peak positions over a well-defined region of pristine graphene, are shown in Figure S2a,b in the Supplementary Information. The G and 2D peaks were observed around 1589 cm^−1^ and 2677 cm^−1^, respectively. Figure 2b shows that the intensity ratio of the 2D and G peaks for pristine graphene was around 1.35, which represents the single layer of graphene. Figure 2c shows the Raman spectroscopy for sample-1 showing the shifts in the G and 2D peak positions for different DUV exposure times. The G peak at 1589 cm^−1^ was shifted to 1595 cm^−1^ and the 2D peak at 2677 cm^−1^ was shifted to 2689 cm^−1^ after DUV irradiation of sample-1 for 60 min. To further confirm the blue shift in the G and 2D peaks, the Raman mapping for G and 2D peak positions of the p-type doped graphene sample under 60 min of DUV exposure in air was carried out as shown in Figure S2c,d in the Supplementary Information. The G and 2D peaks were observed around 1595 cm^−1^ and 2677 cm^−1^, respectively, which confirmed the blue shift after p-type doping. The DUV-irradiation induced an increasing blue shift in the G and 2D peaks with an increase in exposure time which shows that p-type doping increased with increased exposure time. Figure 2b shows the intensity ratios of the 2D and G peaks which decreased from 1.35 to almost 1. This may be because of the increased carrier densities in graphene which has been reported previously [23]. Similarly, the Raman spectrum for the GFET for DUV irradiation with different exposure times for the device (sample-2) in vacuum is presented in Figure 3a. The G and 2D peaks for sample-2 with pristine graphene were observed at 1594 cm^−1^ and 2684 cm^−1^, respectively. The red shift of the G and 2D peak positions of graphene under DUV can be observed in Figure 3c. The G peak was shifted to 1585 cm^−1^ and the 2D peak was shifted to 2670 cm^−1^ after DUV irradiation for 60 min. The red shift in the G and 2D peaks of the n-type doped graphene was further confirmed by the Raman mapping over a well-defined region of n-doped graphene under DUV irradiation with 60 min of exposure time in vacuum, as shown in Figure S2e,f. The red shift of the G and 2D peaks of graphene increased with an increase in the DUV exposure time and is attributed to the induction of the n-type doping effect. Figure 3b shows that the 2D and G intensity ratios also decreased from ~1.45 to 1, which can be explained by an increase in carrier densities.

**Figure 4:**
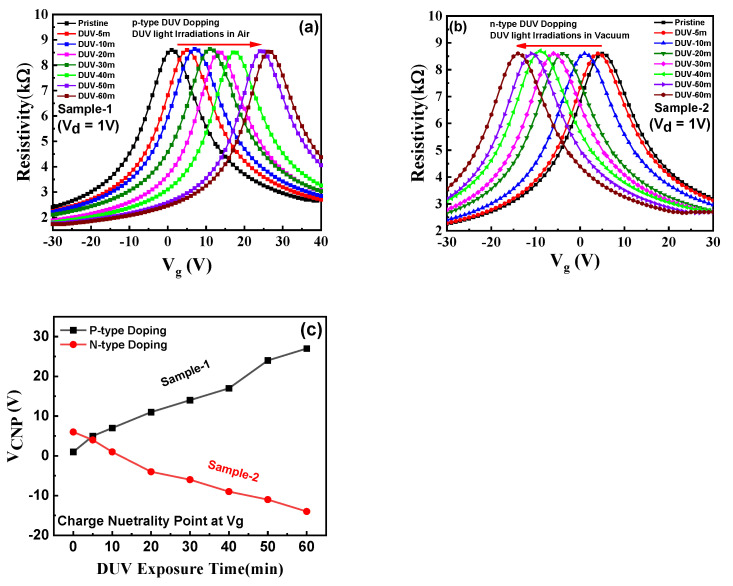
Electrical transport characteristics: (**a**) Resistivity as a function of back-gate voltage (V_g_) for pristine and p-type doping by DUV irradiation in air with different exposure times for sample-1. The p-type doping effect by DUV irradiation in air increased with an increase in exposure time. (**b**) Resistivity as a function of back-gate voltage (V_g_) for pristine and n-type doping by DUV irradiation in vacuum with different exposure times for sample-2. The n-type doping effect by DUV irradiation in vacuum increased with an increase in exposure time, and a saturation point was achieved after 60 min of exposure. (**c**) A shift in the charge neutrality point voltages (V_CNP_) towards positive V_g_ in sample-1 and negative V_g_ in sample-2 because of induced p-type and n-type doping, respectively.

Figure 4a depicts the electric transport characteristics for sample-1 (DUV exposure under air) showing the resistivity as a function of back-gate voltage (V_g_). The charge neutrality point voltage (V_CNP_) in sample-1 shifts towards positive voltages with exposure to DUV irradiation. The V_CNP_ for pristine graphene in sample-1 was near 2 V, which later moved towards a positive V_g_ (27 V for 60 min DUV) with increasing DUV irradiation time, supporting the p-type doping effect. With increase in the DUV exposure time, higher p-type doping in graphene was observed. The shift in V_CNP_ for sample-1 for different exposure times is shown in Figure 4c. It has been reported previously that the p-type doping in graphene under DUV irradiation is induced by the photo-assisted oxidation of the layer [23]. The dissociation of oxygen molecules present in the air occurs under DUV irradiation and creates 2O(^3^P) atoms (O_2_ + hv = 2O(^3^P)) that attach to the most stable adsorption sites of the graphene layer, resulting in p-type doping behavior. The increased intensity of the light can induce a higher shift in V_CNP_ because of increased carrier concentrations. However, there is always a saturation point because only a finite number of charge carriers can be absorbed to the graphene surface [23]. The mobility and bulk charge carrier concentration in the p-type doped graphene sample under 60 min of DUV irradiation in the air were measured by the four-probe Hall measurement system, as shown in Table S1 of Supplementary Information. The Fermi energy (E_F_) level of pristine graphene is normally at equilibrium with the Dirac energy level (E_D_) because of equivalence between the number of electrons and hole concentrations. The Fermi E_F_ level for p-type dope graphene was increased below the Dirac energy level (E_D_) because of increased hole bulk concentrations (4.77 × 10^+19^ [/cm^3^]), as shown in energy band diagram in Figure S3.

The electric transport characteristics for sample-2 (DUV irradiation in vacuum) with resistivity as a function V_g_ are shown in Figure 4b. The V_CNP_ in sample-2 was observed around 5 V which readily shifted towards negative gate voltages (−14 V after 60 min-DUV) with increased treatment time, indicating n-type doping. Sample-2 exhibited a saturation point for n-type doping behavior after 60 min of DUV exposure. Figure 4c shows the shift in CNP positions with different DUV exposure times for both p and n-type doped samples. The exact mechanism underpinning this effect is not fully clear but both a graphene surface and substrate interface charge transfer may play an important role. It has been reported previously that the electrical characteristics of graphene can be tuned by changing the properties of molecules on the surface of graphene and its interface with the substrate [24,25,32]. When DUV is irradiated in vacuum, an electron-hole pair is created by electron trapping adsorbate groups (O^−^ or H_2_O^−^) by hole recombination (h+ + O_2_^−^ = O_2_) and releases electrons. With small changes in carrier mobility and no major changes in the graphene structure, these liberated electrons induce an n-type doping activity in graphene [32]. This does not significantly change the graphene structure; thus, a saturation point was observed after 60 min of DUV exposure in vacuum. Table S1 in Supplementary Information shows the bulk charge carrier concentration and mobility for n-type doped graphene for 60 min of DUV irradiation in vacuum. As the electrons are the majority charge carriers in the n-type dope graphene sample, the E_F_ is increased above E_D_, as shown in the energy band diagram in Figure S3. Figure S4a shows transfer characteristics with resistivity as a function of V_g_ for sample-3 (DUV irradiation in pure O_2_), illustrating the p-type doping in graphene for 60 min of exposure time. The photo-assisted oxidation by DUV irradiation in a pure O_2_ environment induces p-type doping to the graphene. The transfer characteristics of sample-4 (DUV irradiation in pure N_2_) are shown in Figure S4b. The device exhibited n-type doping behavior when exposed to DUV irradiations in a pure N_2_ environment. The shift in V_CNP_ for sample-4 (V_CNP_ = ~−17.8 V) was higher compared with sample-2 (V_CNP_ = ~−13.7 V) for 60 min of DUV exposure time. The N_2_ gas in the presence of DUV irradiation dissociates into atoms which are absorbed on the surface of the graphene. These N_2_ atoms react with the oxygen atoms present on the surface of the graphene and produce O_2_ molecules that are removed [24,25,27,28,29,31,32,39]. The N_2_ atoms under DUV attach to the graphene surface and become the major source of electrons, inducing higher n-type doping. The transfer characteristics for sample-5 were investigated for 60 min DUV in pure N_2_, followed by 60 min irradiation in vacuum, as shown in Figure S4c. The results show that CNP moved toward higher negative gate voltages (V_CNP_ = −24.6 V). Under further DUV exposure in vacuum, the N_2_ atoms attached to the graphene surface induce a further doping effect. Both N_2_ atoms and larger exposure times are responsible for a higher n-type doping effect. Figure S4d shows ∆V_CNP_ (CNP after DUV Doping—CNP before DUV Doping) and V_CNP_ for DUV doping in different irradiation environments. The ∆V_CNP_ shows a higher shift in the CNP position for the air condition in p-type doping, and for pure N_2_ followed by the vacuum condition in n-type doping, for 60 min of DUV exposure time.

**Figure 5:**
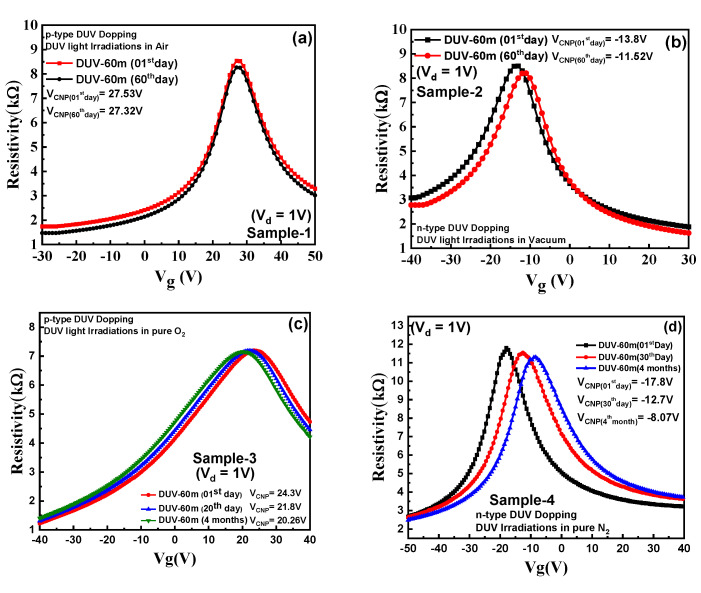

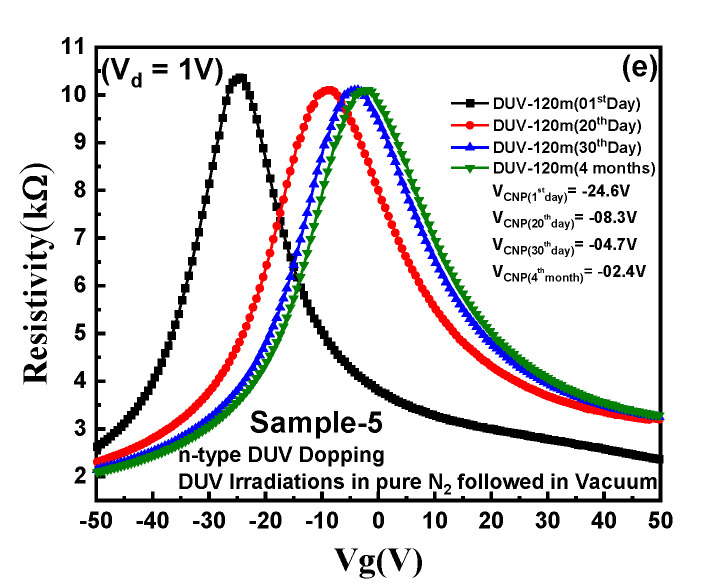
Doping stability: (**a**) Resistivity as a function of V_g_ for sample-1 (DUV in air) for both the 1st and 60th day, exhibiting stable p-doping, confirmed with no shift in CNP. (**b**) Resistivity as a function of V_g_ for n-type doped sample-2 (DUV in vacuum) for both the 1st and 60th day, showing a small de-doping of around 14% over the period. (**c**) Resistivity as a function of V_g_ for sample-3 (DUV in pure O_2_) for the 1st day to 4 months, exhibiting stable p-doping with a minor shift in CNP position. (**d**) Resistivity as a function of Vg for sample-4 (DUV in pure N_2_) for 1st day to 4 months, exhibiting unstable n-doping with large de-doping. (**e**) Resistivity as a function of V_g_ for sample-5 (DUV in pure N_2_ environment followed by DUV in vacuum) for 1st day to 4 months, exhibiting unstable n-doping with a large de-doping effect confirmed by a shift in CNP towards a pristine condition.

The stability of the doping level of graphene is an important factor for its application in electronic devices. Figure 5a shows that sample-1 (DUV in air) exhibited stable p-type doping with a stable CNP over 60 days. The 2O(^3^P) atoms dissociated from oxygen in air under DUV were attached to the most stable bridge sites of graphene, exhibiting stable p-type doping behavior [23]. Figure S5a shows that sample-1 exhibited stable p-type doping at a higher temperature of 200 °C, with different heating times. The doping for sample-2 with n-type doping behavior, over 60 days, is shown in Figure 5b. The device exhibited a de-doping effect of almost 16% by a change in the CNP from V_CNP_ = 13.7 V to 11.3 V. It was predicted that the oxygen from air in the ambient laboratory conditions would attach to the graphene surface over time, inducing a de-doping effect. The oxidation of the graphene layer over the period is the main reason behind the de-doping effect observed in graphene. Figure S5b shows the n-type doping stability of sample-2 with different heating times at a high temperature of 200 °C. Sample-2 exhibited stable doping behavior with a similar CNP at different heating times with a minor increase in carrier mobility. Figure 5c shows the doping stability of sample-3 investigated over 4 months which revealed that a small de-doping of graphene of almost 14% occurred over this period. After the de-doping effect was observed for sample-3, it was predicted that additional O_2_ molecules which were unable to find stable bridge sites would detach from the graphene surface. The doping stability of sample-4 was investigated over 4 months, exhibiting a high de-doping effect of around 54%, as shown in Figure 5d. The dissociated N_2_ atoms attached to the graphene surface, inducing n-type doping, were detached over time because of weak dangling bonds. The oxygen from the ambient air also reacted with the graphene, inducing a large de-doping effect in n-type behavior because of the oxidation. Figure 5e shows the doping stability of sample-5 over 4 months with an almost 90% de-doping effect. As discussed, the N_2_ atoms attached to the graphene surface with weak dangling bonds detached and the O_2_ atoms from the ambient air reacted, resulting in very high de-doping towards a pristine state. Thus, from our studies, we observed that p-type doping stability in sample-1 was very high compared to sample-3, which exhibited a small de-doping effect in sample-3 over time. The n-type doping stability in sample-2 with little de-doping was considerably better compared to sample-4 and sample-5 which showed a large de-doping effect over time.

**Figure 6:**
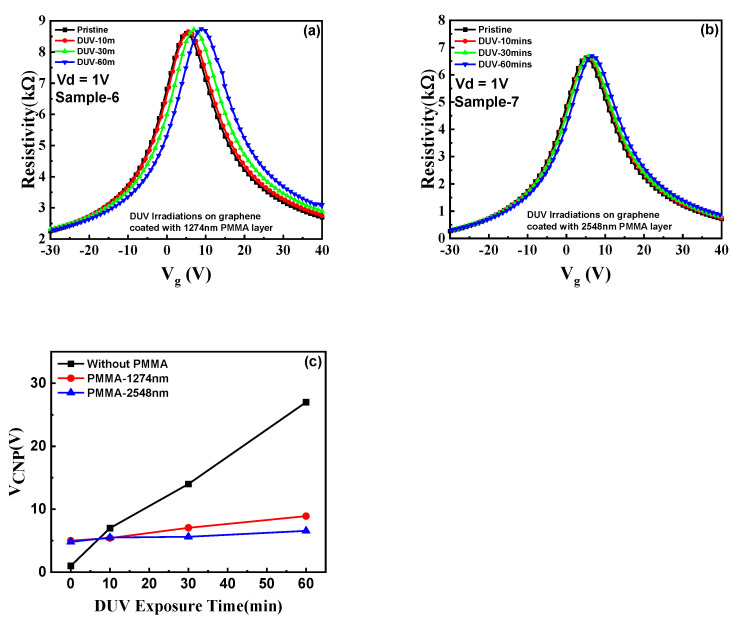
DUV effect on PMMA coated graphene: (**a**) Resistivity as a function of back-gate voltage (V_g_) for sample-6 (1274nm PMMA layer coated graphene) showing a minor shift in CNP with increased DUV exposure time. (**b**) Resistivity as a function of back-gate voltage (V_g_) for sample-7 (3548 nm PMMA layer coated graphene), exhibiting a negligible shift in CNP with increased DUV exposure time. (**c**) A shift in the charge neutrality point voltages (V_CNP_) for devices coated without a PMMA layer, 1274 nm PMMA layer, and 2548 nm PMMA layer. The device with a 2548 nm PMMA- coated layer exhibited a negligible shift in the CNP position, confirming no DUV effect on graphene.

Furthermore, the effect of DUV light irradiation on graphene, covered with PMMA, was investigated by assessing the transfer characteristics of the device for different DUV treatment times, as shown in Figure 6. The graphene (sample-6) was coated with PMMA with a thickness of 1274 nm and a controlled spin-coating speed of 4000 rpm [40,41,42,43]. The device was then exposed to DUV irradiation for different time intervals and transfer characteristics of the device were studied, as shown in Figure 6a. The GFET coated with a single layer of PMMA (1274 nm) exhibited a small change in the CNP position, as compared to devices exposed to DUV irradiation without any PMMA coating for similar DUV exposure times. To further study the DUV effect, two layers of PMMA, with a thickness of around 2548 nm, were coated by a spin coating process over the GFET (sample-7). Figure 6b shows the transfer characteristics of the device with two PMMA layers, exhibiting no change in the CNP, a stable V_CNP_, similar to pristine graphene. No change in electronic properties and the doping effect in graphene by DUV irradiation were observed for the device coated with a thick PMMA layer. As previously reported, the transmission of light through the PMMA layer decreases when the thickness of the film is increased [42,43]. The results showed that the DUV light was absorbed into the thick PMMA layer, and the graphene underneath was not influenced by the DUV light irradiation. Figure 6c shows the comparison between different V_CNP_ positions for pristine graphene, single layer PMMA (1274 nm), and double-layer PMMA (2548 nm) coated graphene, respectively. It can be readily observed that sample-7, with a thicker layer of PMMA (2548 nm), showed a negligible shift in the CNP position for different DUV exposure times. Figure S6a shows the Raman spectrum of the graphene sample-7 for different exposure times. No shift in the 2D and G peaks of the graphene was observed for the device as compared to the pristine graphene. The transfer characteristics and Raman spectroscopy of the device implies that PMMA can be used as a capping layer to protect the graphene from a light-induced effect on its structural and electronic properties.

**Figure 7:**
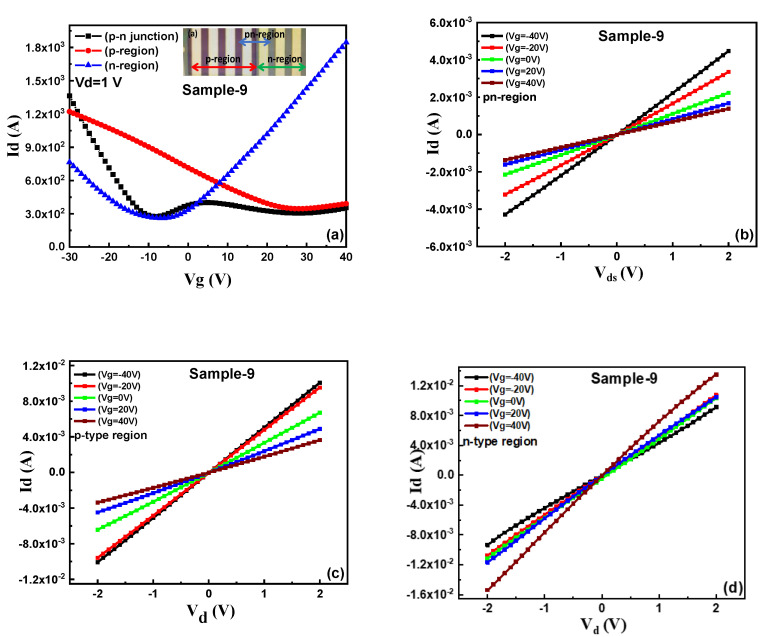
pn-junction: (**a**) Drain current (I_d_), as a function of gate voltage for sample-9, showing transfer characteristics of the pn-junction region (black curve), p-region (red curve), and n-region (blue-curve). The pn-junction is a combination of p and n-type doped regions in a lateral channel of single-layer CVD graphene. (**b**) I_d_-V_d_ characteristics of the pn-junction region in sample-9 at different gate voltages. (**c**) I_d_-V_d_ characteristics of the p-region in sample-9, at different gate voltages. (**d**) I_d_-V_d_ characteristics of the n-region in sample-9, at different gate voltages.

The DUV induced n and p-type doping are reversible under controlled conditions [20,21,22,25,30]. Figure S7a shows the transfer characteristics of sample-8 to investigate the reversible nature of DUV-induced doping. The device was initially n-type doped by 60 min of DUV irradiation in vacuum. Further, the device was exposed to DUV irradiation in air which exhibited counter doping and high p-type doping which was induced after 120 min exposure. A pn-junction on a single channel of CUV grown graphene was successfully fabricated, by utilizing the DUV irradiation-induced doping effect. The device (sample-9) was n-type doped with DUV irradiation in vacuum and was coated by PMMA. Then a specific graphene channel region was patterned by e-beam lithography and PMMA was removed with a methyl isobutyl ketone (MIBK) solution. The device was further exposed to DUV light irradiation in air to achieve p-type doping in the uncovered graphene region. The n-type doped region was protected by the PMMA layer and stable p-type doping was induced in the uncovered graphene area by DUV light irradiation in air. Figure 7a shows the transfer characteristics with I_d_ as a function of V_g_ in sample-10 for the pn-junction, p, and n regions of the device. An optical image of the device with defined n, p, and pn-regions is shown in the inset of Figure 7a. The blue curve shows the transfer characteristics of the PMMA-coated, n-typed doped region of the graphene with a V_CNP_ of around −8 V. The red transfer curve exhibited the counter doped p-type behavior of the graphene with a V_CNP_ position at around 30 V f. On investigation of the transfer characteristics of the pn-region, it exhibited two distinct CNPs at negative and positive V_g_, each corresponding to the n-type and p-type doping effect in graphene, shown by the black transfer curve in Figure 7a. The Fermi level of graphene was raised by DUV irradiation in air and reduced by irradiation in vacuum compared to the Fermi level of pristine graphene [22,23,24]. Thus, these results imply that the difference in the Fermi levels of graphene for the n and p-type regions can be used to construct a graphene pn-junction on the single layer of CVD-grown graphene [22,23,24,25]. The I_d_-V_d_ characteristics of the n, p, and pn-regions at different V_g_ with I_d_ sweep from −2 V to 2 V, are shown in Figure 7b–d respectively. Figure 7b,c show typical I–V behavior with an increase in I_d_ on positive gate voltages for n-type and a decrease in I_d_ on positive gate voltages for p-type regions, respectively. Figure 7d shows that the pn-junction also exhibited linear behavior because of the gapless band structure of the graphene [22,23,24]. The I_d_-V_d_ characteristics of the n, p, and pn-regions at different V_g_ with I_d_ sweep from −2 V to 2 V, are shown in Figure 7b–d, respectively. Figure 7b,c show typical I-V behavior with an increase in I_d_ on positive gate voltages for n-type and a decrease in I_d_ on positive gate voltages for p-type regions, respectively. Figure 7d shows that the pn-junction also exhibited linear behavior because of the gapless band structure of the graphene [22,23,24,25]. The results for all the graphene regions show linear behavior as in previous studies [22,23,24,25]. 

**Figure 8:**
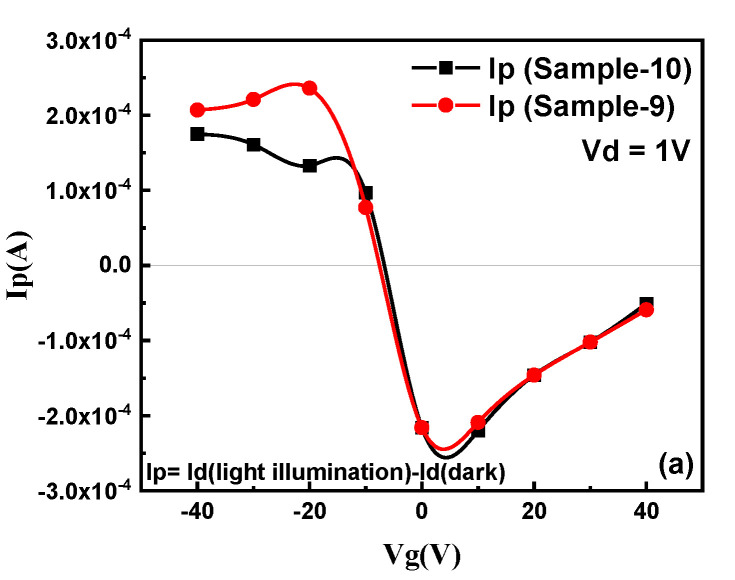

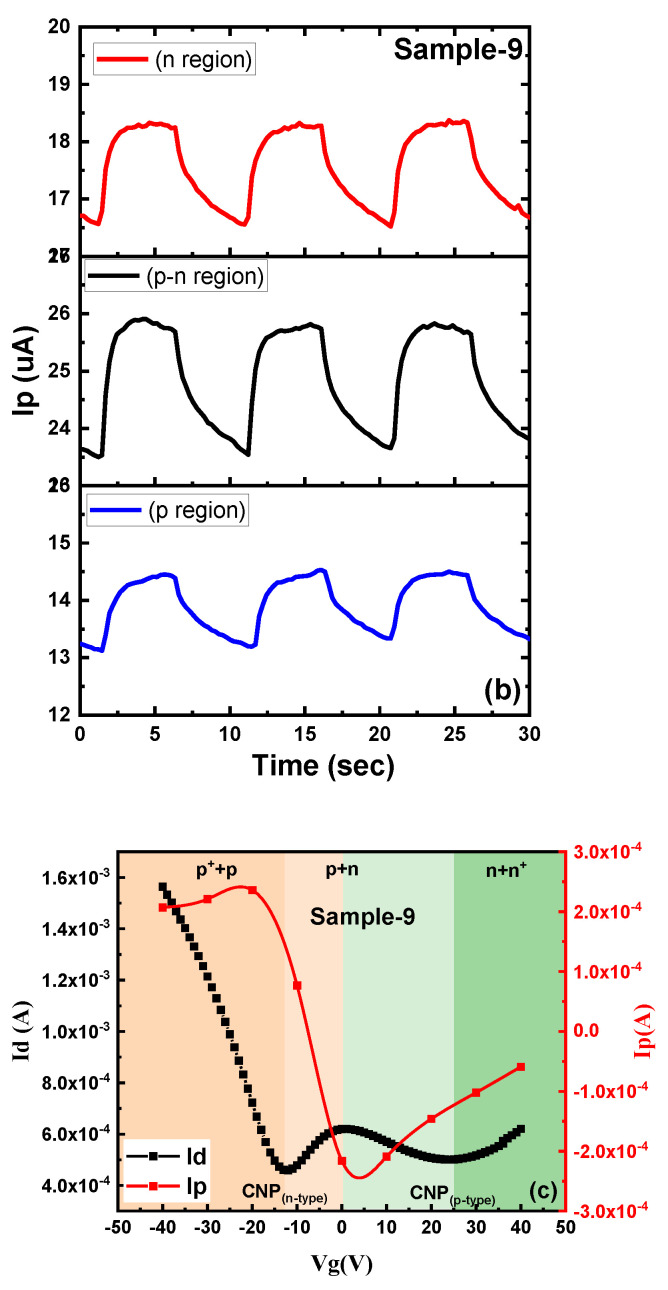
Photo-current (I_p_): (**a**) V_g_ dependent Ip measured for fabricated pn-junction devices (sample-9 and sample-10). (**b**) I_p_ as a function of time with a bias voltage Vd of 1V in the dark and illuminated states during the measurement. The I_p_ for the pn-region is readily enhanced because of photo-generated electron-hole pairs in the channel. (**c**) V_g_ dependent I_d_ and I_p_ for the fabricated pn-junction device (sample-9), for different regions of the device.

The photocurrent (I_p_) (the difference of I_d_ under light and I_d_ without light illumination) as a function of V_g_ was investigated for graphene pn-junction devices using an ultraviolet light (λ = 365 nm and 350 µWcm^−2^ intensity) source (Mightex P/N WLS-22-A light source) as shown in Figure 8a. The Ip and time response for the n, p, and pn-junction regions for sample-10, at V_d_ of 1 V, is shown in Figure 8b. The I_p_ for the pn-junction was readily enhanced as compared to the p and n region of the graphene because of an increase in the photocarrier’s generated built-in potential created by the formation of the pn-junction [35]. Figure 8c shows the comparison of Ip to the transfer curve (Id) of the device (sample-10) as a function of V_g_. The Ip curve depicts a positive photocurrent at negative V_g_ which undergoes a sign reversal and becomes negative for positive V_g_, which is consistent with previously reported studies [33,34,35,38]. This shows that both the magnitude and polarity of I_p_ can be modulated by V_g_ [33,34,35]. The photocurrent corresponds to three different gate-controlled regions in the device channel that are p^+^+p, p+n, and n+n^+^, respectively [33,34,35]. The two charge neutrality points (CNP_n-type_ and CNP_p-type_) formed by the hole and electron conduction regions confirm the formation of the pn-junction in the graphene single channel [35]. The built-in electric potential induced by the formation of the pn-junction governs the mobility of charge carriers and can be controlled by a gate-modulated shift in Fermi levels of the two differently doped regions [33]. The light-induced carrier density in the graphene is considerably less; thus, high carrier mobility in the channel is considered responsible for the resulting photocurrent [34,35]. In addition, the photo-generated charge carriers in Si/SiO_2_ led to an increase in conductivity, and the electric field effect in the graphene was enhanced due to applied gate voltages resulting in increased mobility, as also observed in other semiconducting 2D materials [35,37,38]. A gate-dependent Fermi level shift between p- and n-type doped regions of the sheet is considered to explain the photocurrent generation mechanism [33]. Thus, the electrostatically induced carrier density can be changed with different V_g_ and can modulate the polarity and magnitude of Ip with applied gate voltages [33,34,35,38]. The maximum I_p_ was observed around a V_g_ of −20 V outside the pn-junction region of the device which usually has the maximum potential difference of the p and n regions. This can be explained by the fact that light was illuminated on the whole device which includes not only the p+n region but also the metal and interface junction in p^+^+p and n+n^+^ regions of the graphene [33,34,35,38]. Thus, these results confirm that the photocurrent was determined by the mobility of the charge carriers for the whole graphene channel region under light illumination, which has also been reported in previous studies [34,35]. 

## 4. Conclusions

In this study, we thoroughly investigated the DUV-irradiation-induced doping effect in CVD-grown graphene, under controlled irradiation environments (air, vacuum, pure O_2_, and pure N_2_ environments, and graphene covered with PMMA). The shift in G and 2D peaks of Raman spectra towards higher wavenumbers and the shift of CNP towards positive V_g_ values in transfer characteristics confirmed the p-type doping for graphene devices with an increase in DUV exposure time in air and pure O_2_ environments. The p-type doped graphene exhibited high stability over time with a minor de-doping (14%) effect in the pure O_2_ environment. The shift in G and 2D peaks towards smaller wavenumbers in Raman spectra and the CNP shift towards negative gate voltages in transfer characteristics confirmed the n-type doping in graphene exposed to DUV irradiation in vacuum and pure N_2_ environments. The n-type doping with a small de-doping (16%) effect for the device exposed in vacuum was considerably better compared to highly unstable n-type doping with a large de-doping (54% and 90%) effect over time for devices exposed to DUV in pure N_2_ and pure N_2_ followed by a vacuum environment. The thick PMMA (2548 nm) layers reduced the DUV irradiation effect on graphene by increased light absorbance and reduced light transmittance. A lateral pn-junction in a single graphene channel was fabricated in a controlled DUV irradiation environment and specified doping in selected regions with and without PMMA coating. The back gate-dependent transfer and Id-Vd characteristics for n, p, and pn regions of the device validate the process to be a suitable technique to alter the electronic properties of graphene, without varying the physical and chemical properties of graphene. The results of photo-response current measurement as a function of back-gate voltage showed increased photo-response of the device because of increased field-effect mobility in the graphene channel. This study has presented a possible photo-assisted technique under defined conditions for the modification of the electronic properties of graphene for potential future application in electronic and optoelectronic devices.

## Data Availability

Not applicable.

## Supplementary Materials

The following are available online at https://www.mdpi.com/article/10.3390/nano11113003/s1, Figure S1 Raman spectroscopy; (a) The Raman spectroscopy analysis for sample-1 with different exposure time to induce p-type doping in graphene by DUV irradiations in air, with different exposure times. (b) The Raman spectroscopy analysis for sample-2 with different exposure time to induce p-type doping in graphene by DUV irradiations in vacuum, with different exposure times; Figure S2 Raman Mapping: (a,b) The Raman mapping for G and 2D peak positions for well-defined area of pristine graphene. (c,d) The Raman mapping for G and 2D peak positions for well-defined area of p-type doped graphene under DUV irradiations in air for 60 min. (e,f) The Raman mapping for G and 2D peak positions for well-defined area of n-type doped graphene under DUV irradiations in vacuum for 60 min; Figure S3: Energy Band Diagram: Energy band diagram for pristine, n-type and p-type dopes graphene. Depending on the majority charge carrier concentration the E_F_ is increased or decreased above or below E_D_ for n and p type doped samples, respectively; Figure S4 Transfer characteristics and a shift in charge neutrality points (CNP): (a) Transfer characteristic with resistivity as a function of V_g_ for sample-3 (DUV irradiation in pure O_2_) illustrating the p-type doping in graphene, for 60 min of exposure time. (b) Transfer characteristics of sample-4 (DUV irradiations in pure N_2_), exhibiting n-type doping behavior when exposed to DUV irradiations in pure N_2_ environment for 60 min. (c) Transfer characteristics for sample-5 (60 min DUV in pure N_2_, followed by 60 min irradiations in vacuum), exhibiting a high n-type doping effect. (d) ∆V_CNP_ (CNP after DUV Doping – CNP Before DUV Doping) and V_CNP_ for DUV doping under different irradiation environments, showing a higher shift in the CNP position under air conditions in p-type doping and pure N_2_, followed by vacuum condition in n-type doping, for 60 min of DUV exposure time; Figure S5 Stability with heating time: (a) DUV irradiations in air induced doping state of the device (sample-1) with different heating times, from 15 min to 120 min, at a temperature of a 200 °C. (b) DUV irradiations in vacuum induced doping state of the device (sample-2) with different heating times, from 15 min to 120 min, at a temperature of a 200 °C. Both devices exhibit a stable doping state over the different heating times; Figure S6 Raman for PMMA coated graphene: (a) Raman spectrum of graphene-coated 2548 nm thick PMMA and exposed to DUV for different intervals of time, up to 60 min. No shift in the 2D and G peaks of graphene was observed, as compared to pristine graphene for different exposure times; Figure S7 Transfer characteristics for reversible DUV-Doping: (a) Transfer characteristics of sample-8 to investigate the reversible nature of DUV induced doping. The device was initially n-type doped, by 60 min of DUV irradiations in vacuum. Further, the device was exposed to DUV irradiation in air, which exhibits counter doping and high p-type doping was induced, after 120 min exposure; Table S1: Hall measurement characteristics.
