# Supplementary material for: Deep-Ultraviolet (DUV)-Induced Doping in Single Channel Graphene for Pn-Junction
Source: Nanomaterials (Basel). 2021 Nov 9;11(11):3003. doi: 10.3390/nano11113003 (PMC8623685; doi:10.3390/nano11113003)
Supplement: Supplementary file 1 [file nanomaterials-11-03003-s001.zip › nanomaterials-1396501-supplementary.pdf]

Supplementary Material

Deep-Ultraviolet (DUV)-Induced Doping in Single Channel Graphene for Pn-Junction

Asif Ali [1], So-Young Kim [2], Muhammad Hussain [1], Syed Hassan Abbas Jaffery [1], Ghulam Dastgeer [3], Sajjad Hussain [1], Bach Thi Phuong Anh [1], Jonghwa Eom [3], Byoung Hun Lee [2] and Jongwan Jung [1,*]

[1] HMC (Hybrid Materials Center) and Department of Nanotechnology and Advanced Materials Engineering, Sejong University, Seoul 05006, Korea; asifmju@gmail.com (A.A.); mhussain@sju.ac.kr (M.H.);
hassam@sju.ac.kr (S.H.A.J.); shussainawan@gmail.com (S.H.);
bachthiphuonganh@gmail.com (B.T.P.A.)

[2] Center for Semiconductor Technology Convergence, Department of Electrical Engineering, Pohang
University of Science and Technology, Cheongam-ro 77, Nam-gu, Pohang 37673, Korea; una0918@postech.ac.kr (S.-Y.K.); bhlee1@postech.ac.kr (B.H.L.)

[3] Department of Physics & Astronomy and Graphene Research Institute-Texas Photonics Center International Research Center (GRI–TPC IRC), Sejong University, Seoul 05006, Korea; gdastgeer@sejong.ac.kr (G.D.); eom@sejong.ac.kr (J.E.)

* Correspondence: jwjung@sejong.ac.kr

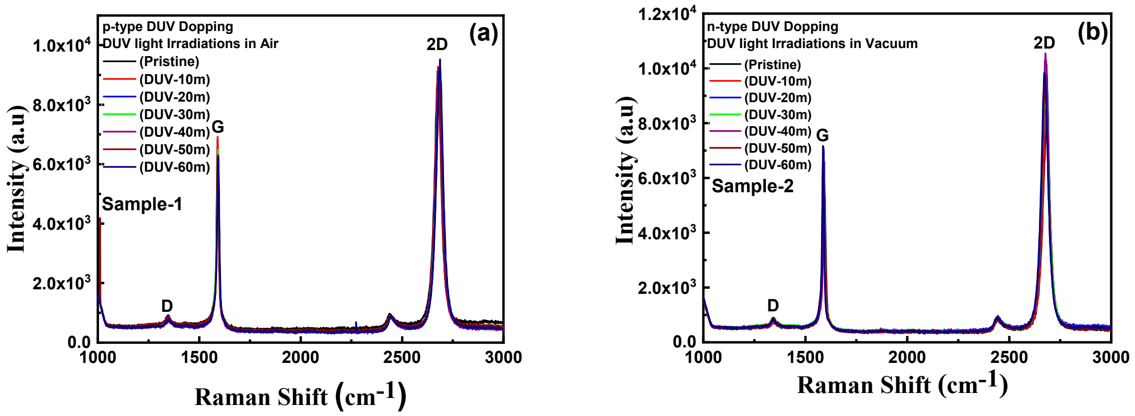

Figure S1: Raman spectroscopy; (a) The Raman spectroscopy analysis for sample-1 with different exposure time to induce p-type doping in graphene by DUV irradiations in air, with different exposure times. **(b)** The Raman spectroscopy analysis for sample-2 with different exposure time to induce p-type doping in graphene by DUV irradiations in vacuum, with different exposure times.

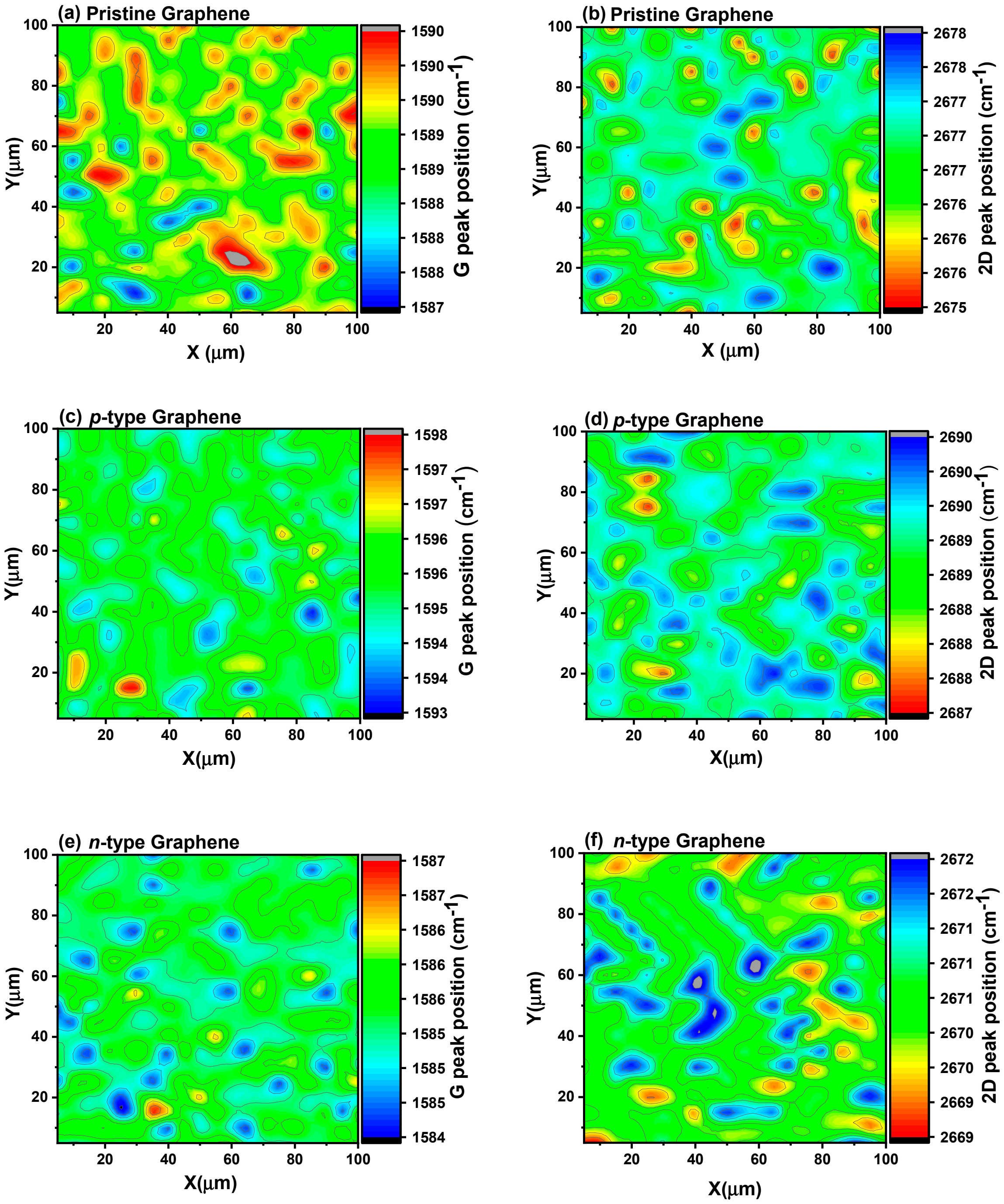

Figure S2 Raman Mapping: (a, b) The Raman mapping for G and 2D peak positions for well-defined area of pristine graphene. **(c, d)** The Raman mapping for G and 2D peak positions for well-defined area of p-type doped graphene under DUV irradiations in air for 60mins. **(e, f)** The Raman mapping for G and 2D peak positions for well-defined area of n-type doped graphene under DUV irradiations in vacuum for 60mins.

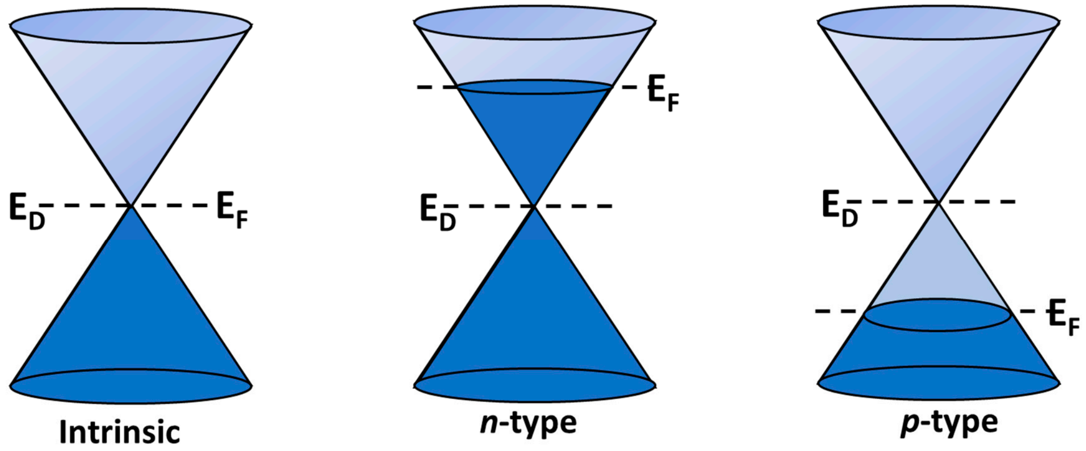

Figure S3 Energy Band Diagram: Energy band diagram for pristine, n-type and p-type dopes graphene. Depending on the majority charge carrier concentration the E_F is increased or decreased above or below E_D for n and p type doped samples, respectively.

Table-S1: Hall measurement characteristics

Characteristics	*p-type Graphene*	*n-type Graphene*
Bulk Carrier Concentration	***4.77 x 10^{+19} [/cm^3]***	***-3.426 x 10^{+15} [/cm^3]***
Sheet Carrier Concentration	***4.808 x 10^{+12} [/cm^2]***	***-3.413 x 10^{+8} [/cm^2]***
Mobility	***10.198 x 10^{+3} [cm^2/Vs]***	***7.006 x 10^{+3} [cm^2/Vs]***
Sheet Resistance	***1.084 x 10^{+3} [Ω/square]***	***2.601 x 10^{+3} [Ω/square]***
Average Hall Coefficient	***1.298 x 10^{-1} [cm^3/C]***	***1.822 x 10^{-3} [cm^3/C]***

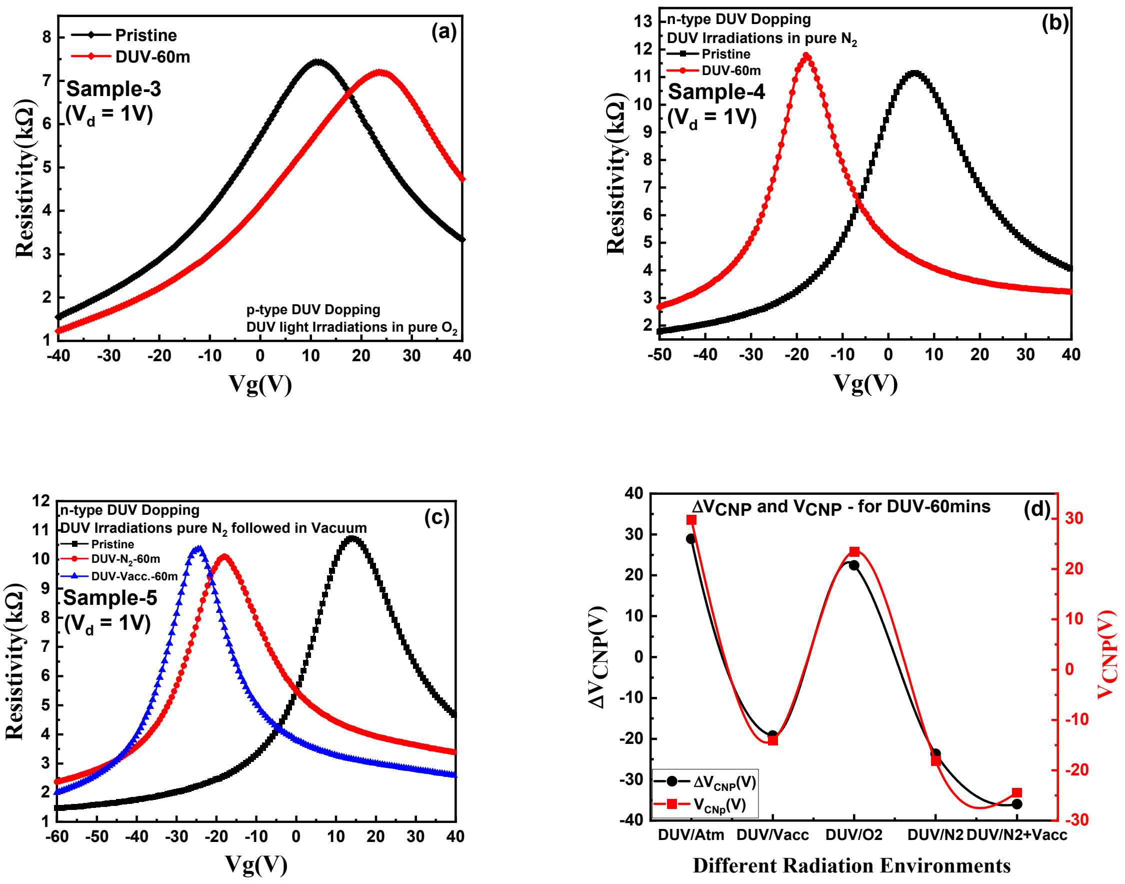

Figure S4 Transfer characteristics and a shift in charge neutrality points (CNP): (a) Transfer characteristic with resistivity as a function of V_g for sample-3 (DUV irradiation in pure O_2) illustrating the p-type doping in graphene, for 60 mins of exposure time. **(b)** Transfer characteristics of sample-4 (DUV irradiations in pure N_2), exhibiting n-type doping behavior when exposed to DUV irradiations in pure N_2 environment for 60mins. **(c)** Transfer characteristics for sample-5 (60 min DUV in pure N_2, followed by 60 mins irradiations in vacuum), exhibiting a high n-type doping effect. **(d)** ΔV_{CNP} (CNP after DUV Doping – CNP Before DUV Doping) and V_{CNP} for DUV doping under different irradiation environments, showing a higher shift in the CNP position under air conditions in p-type doping and pure N_2, followed by vacuum condition in n-type doping, for 60 mins of DUV exposure time.

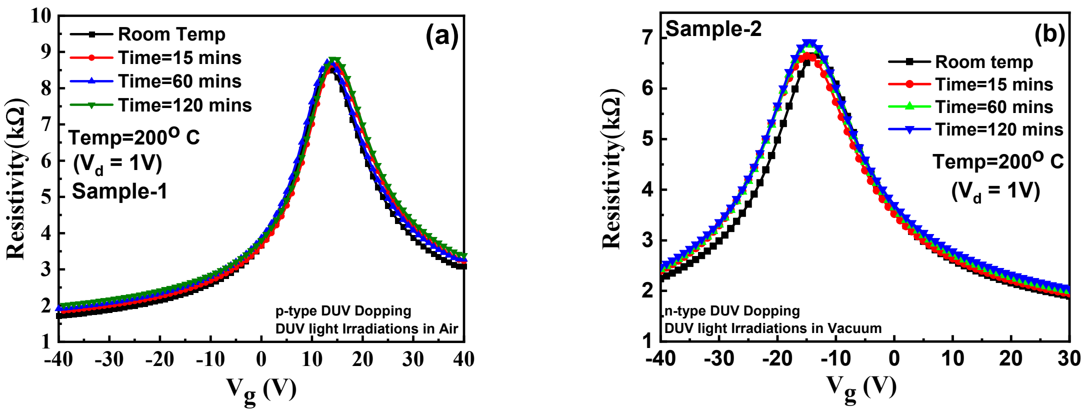

Figure S5 Stability with heating time: (a) DUV irradiations in air induced doping state of the device (sample-1) with different heating times, from 15 mins to 120 mins, at a temperature of a 200°C. **(b)** DUV irradiations in vacuum induced doping state of the device (sample-2) with different heating times, from 15 mins to 120 mins, at a temperature of a 200°C. Both devices exhibit a stable doping state over the different heating times.

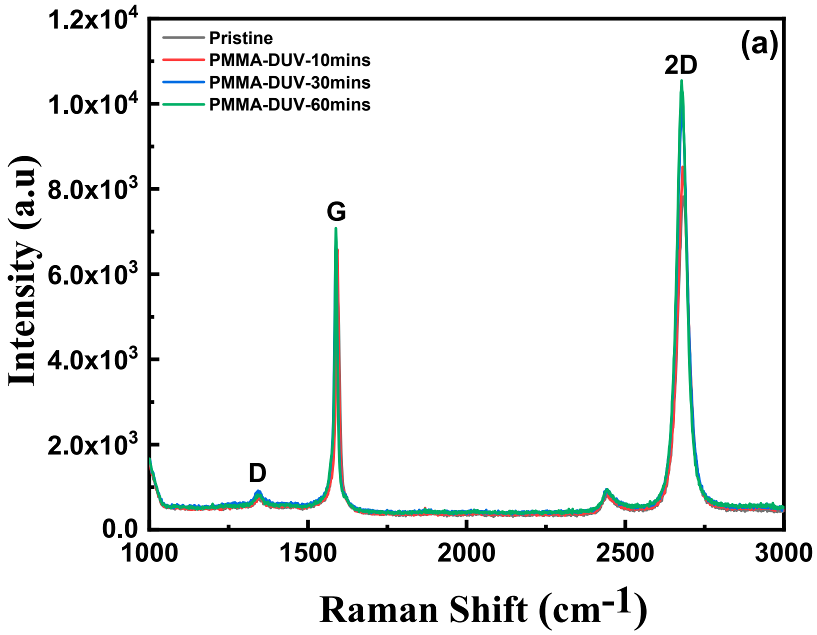

Figure S6 Raman for PMMA coated graphene: (a) Raman spectrum of graphene-coated 2548nm thick PMMA and exposed to DUV for different intervals of time, up to 60 mins. No shift in the 2D and G peaks of graphene was observed, as compared to pristine graphene for different exposure times.

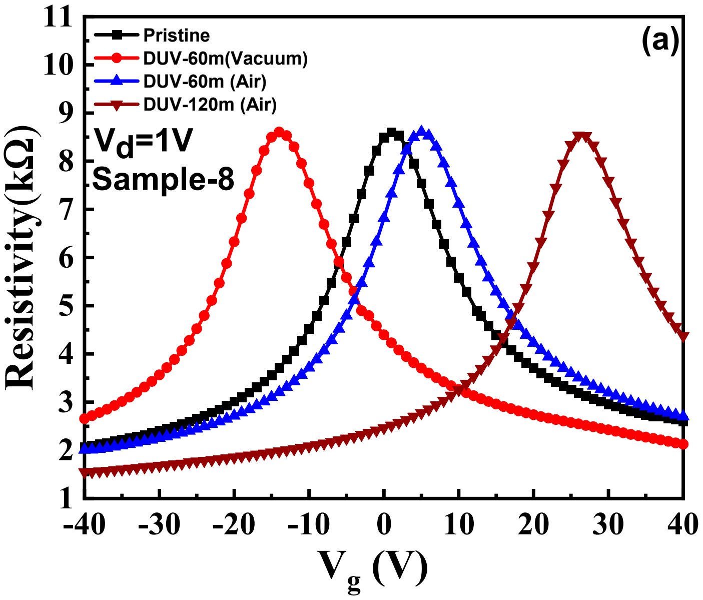

Figure S7 Transfer characteristics for reversible DUV-Doping: (a) Transfer characteristics of sample-8 to investigate the reversible nature of DUV induced doping. The device was initially n-type doped, by 60 mins of DUV irradiations in vacuum. Further, the device was exposed to DUV irradiation in air, which exhibits counter doping and high p-type doping was induced, after 120 mins exposure.
